# SNF8, a member of the ESCRT-II complex, interacts with TRPC6 and enhances its channel activity

**DOI:** 10.1186/1471-2121-13-33

**Published:** 2012-11-21

**Authors:** Robert Carrasquillo, Dequan Tian, Sneha Krishna, Martin R Pollak, Anna Greka, Johannes Schlöndorff

**Affiliations:** 1 Division of Nephrology, Beth Israel Deaconess Medical Center, Research North 304B, 99 Brookline Ave, Boston, MA, 02215, USA; 2 Department of Medicine, Nephrology Division, Massachusetts General Hospital, 149 13th Street, Room 8.102, Boston, MA, 02114, USA; 3 Harvard Medical School, Boston, MA, 02115, USA

**Keywords:** Transient receptor potential, Calcium channel, Protein-protein interaction, Calcineurin-NFAT signaling

## Abstract

**Background:**

Transient receptor potential canonical (TRPC) channels are non-selective cation channels involved in receptor-mediated calcium signaling in diverse cells and tissues. The canonical transient receptor potential 6 (TRPC6) has been implicated in several pathological processes, including focal segmental glomerulosclerosis (FSGS), cardiac hypertrophy, and pulmonary hypertension. The two large cytoplasmic segments of the cation channel play a critical role in the proper regulation of channel activity, and are involved in several protein-protein interactions.

**Results:**

Here we report that SNF8, a component of the endosomal sorting complex for transport-II (ESCRT-II) complex, interacts with TRPC6. The interaction was initially observed in a yeast two-hybrid screen using the amino-terminal cytoplasmic domain of TRPC6 as bait, and confirmed by co-immunoprecipitation from eukaryotic cell extracts. The amino-terminal 107 amino acids are necessary and sufficient for the interaction. Overexpression of SNF8 enhances both wild-type and gain-of-function mutant TRPC6-mediated whole-cell currents in HEK293T cells. Furthermore, activation of NFAT-mediated transcription by gain-of-function mutants is enhanced by overexpression of SNF8, and partially inhibited by RNAi mediated knockdown of SNF8. Although the ESCRT-II complex functions in the endocytosis and lysosomal degradation of transmembrane proteins, SNF8 overexpression does not alter the amount of TRPC6 present on the cell surface.

**Conclusion:**

SNF8 is novel binding partner of TRPC6, binding to the amino-terminal cytoplasmic domain of the channel. Modulating SNF8 expression levels alters the TRPC6 channel current and can modulate activation of NFAT-mediated transcription downstream of gain-of-function mutant TRPC6. Taken together, these results identify SNF8 as a novel regulator of TRPC6.

## Background

Transient receptor potential canonical channel 6 (TRPC6) is one of seven members of the canonical transient receptor potential (TRPC) family of calcium-permeable cation channels that allow for an increase in intracellular calcium following activation of G-protein coupled receptors and receptor tyrosine kinases [1]. TRPC channel subunits assemble to form homo- and/or hetero-tetramers [2]. Each TRPC subunit contains intracellular amino and carboxyl-terminal domains flanking six transmembrane domains; the intracellular sequences host canonical protein-protein interaction domains, including ankyrin-like repeats and a coiled-coil region in the amino-terminal cytoplasmic domain, and carboxyl-terminal TRP box, CIRB (calmodulin/IP3 receptor binding) sequence, and a coiled-coil region [3]. Multiple proteins have been shown to bind to TRPC6, including BK_Ca_[4], calmodulin [5-7], FKBP12 [8], Fyn [9], IP3 receptor [7,10], MxA [11], the Na+/K+-ATPase pump [12], PLCγ [13], podocin [14,15], PTEN [16], and RNF24 [17]. In addition, TRPC6 channel activity can be modulated by tyrosine [9,13] and serine/threonine phosphorylation [18-21], and by binding of phosphoinositides [6,22,23]. However, how these various post-translational modifications and interacting partners ultimately alter channel activity has not been fully elucidated.

TRPC6 has been implicated in several physiological and pathophysiological functions in the kidney, heart and vasculature. Mutations in TRPC6 are a cause of familial, autosomal-dominant, adult-onset focal segmental glomerulosclerosis (FSGS), a form of proteinuric renal disease [15,24], and increased TRPC6 expression has been observed in acquired proteinuric kidney disease [25,26]. In contrast, TRPC6-deficient mice show mild protection against angiotensin II mediated glomerular damage [27]. The majority of FSGS-associated mutations appear to be gain-of-function, with increased channel current amplitudes and reduced current decay having been reported [15,24,28,29]. As all currently identified FSGS disease-associated TRPC6 mutations map to the intracellular domains of the channel, it has been hypothesized that these mutations may act by disrupting the binding of regulatory proteins to TRPC6, with the slit diaphragm protein nephrin proposed as one such protein [13]. How enhanced TRPC6 activity leads to renal disease, though, remains unknown. In contrast, in the heart upregulation of TRPC6 has been linked to the development of pathologic cardiac hypertrophy through activation of the calcineurin-NFAT pathway [30,31]. Finally, examinations of TRPC6-deficient mice have suggested roles in hypoxic pulmonary vasoconstriction [32], the development of pulmonary edema in response to ischemia-reperfusion injury [33], and platelet function [34].

To discover novel binding partners of TRPC6, we performed a yeast two-hybrid screen using the amino-terminal domain of TRPC6 as bait and isolated SNF8 as a TRPC6 binding partner. SNF8 is the mammalian counterpart to yeast VPS22, a component of the ESCRT-II endosomal trafficking complex required for the endocytosis and lysosomal degradation of transmembrane proteins [35-37]. The SNF8-TRPC6 interaction was mapped to the first 107 residues of the N-terminus of TRPC6, and was confirmed by co-immunoprecipitation as well as immunofluorescence microscopy. Additionally, overexpression and RNAi-mediated knockdown of SNF8 in tissue culture reveals a modulatory effect on wild-type and mutant TRPC6 channel activity as evidenced by whole-cell electrophysiology studies and transcriptional luciferase reporter assays.

## Results

### SNF8 binds to the amino-terminus of TRPC6 in the Yeast Two-Hybrid System

**Figure 1:**
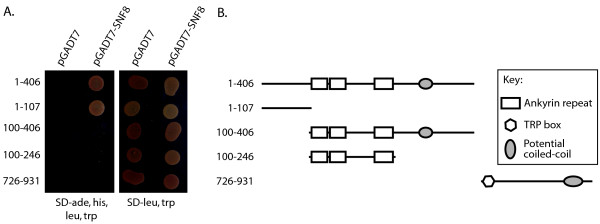
**SNF8 binding to TRPC6 in the two-hybrid assay. A**. Yeast carrying the pGBKT7 plasmid encoding for the Gal4 DNA binding domain fused to the indicated TRPC6 amino acids, and either pGADT7 (encoding for the GAL4 activation domain alone) or pGADT7-SNF8 (expressing full-length SNF8 fused to the GAL4 activation domain) were grown on high stringency selective media, SD-ade, his, leu, trp (left panel), to assess for protein-protein interaction, or on SD-leu, trp (right panel) as a control. **B**. Schematic of TRPC6 structure indicating the location of various structural domains relative to the TRPC6 constructs used in the yeast-two hybrid assay.

The amino-terminal cytoplasmic domain of human TRPC6 (amino acids 1-406) was used as bait in a yeast two-hybrid screen to identify potential TRPC6 interacting proteins in a human kidney cDNA library. On the basis of both nutritional selection and β-galactosidase activity, 10 clones of SNF8 (also known as EAP30 and VPS22) were isolated; all clones included the entire coding sequence of SNF8 and varying lengths of 5′ UTR. Full-length SNF8 fused to the Gal4 activation domain was able to interact with the amino-terminal cytoplasmic domain, but not the carboxyl-terminal cytoplasmic domain of TRPC6 in the yeast two-hybrid system as assessed by growth on histidine and adenine free media (Figure 1) and β-galactosidase activity (data not shown). Using truncation mutants of the amino-terminal of TRPC6, it was found that amino acids 1-107, containing the sequence prior to the ankyrin repeats, are necessary and sufficient for this interaction (Figure 1).

### TRPC6 and SNF8 interact in mammalian cells

**Figure 2:**
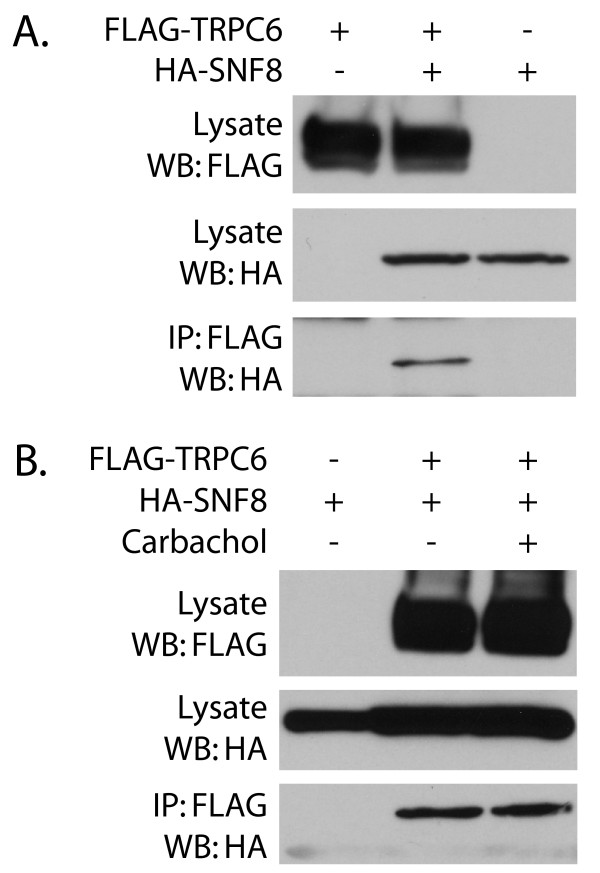
**Binding of TRPC6 to SNF8 in 293T cells. A**. M1R cells expressing FLAG-tagged TRPC6 under a tetracycline inducible promoter were transfected with HA-tagged SNF8 or control vector, followed by culture in the absence or presence of 1 μg/ml tetracycline to induce the expression of TRPC6. Cell lysates were either directly analyzed for TRPC6 and SNF8 expression by Western blot (top and middle panel, respectively), or incubated with FLAG M2 affinity beads to immunoprecipitate TRPC6. HA-tagged SNF8 was detected bound to the affinity beads when expressed in the presence of TRPC6 (bottom panel). **B**. Cells transfected with HA-tagged SNF8 and induced to express TRPC6 as indicated were treated with or without 100 μM carbachol for 5 minutes prior to lysis and immunoprecipitation as in (**A**) above.

To confirm that the interaction between TRPC6 and SNF8 can occur in a cellular context, cells stably expressing FLAG-tagged TRPC6 under a tetracycline inducible promoter were transfected with HA-tagged SNF8. TRPC6 was immunoprecipitated with the FLAG M2 monoclonal antibody, and the presence of SNF8 in the isolated immune complexes was assessed by Western blot using an anti-HA antibody (Figure 2A). HA-SNF8 was detected in the immunoprecipitated material only when co-expressed with FLAG-TRPC6. We have not been able to confirm the interaction by co-immunoprecipitation of endogenous SNF8 and TRPC6, but this may be due at least in part due to the limited availability of high quality antibodies to detect endogenous SNF8 (data now shown) or a relatively low affinity interaction between the two proteins.

To assess if the interaction between the two proteins might be influenced by TRPC6 channel activation, cells co-expressing both proteins were treated with or without carbachol to activate TRPC6 prior to cell lysis and immunoprecipitation. No notable difference in the amount of SNF8 in the immune complexes was seen upon carbachol stimulation (Figure 2B). This suggests that channel activity or calcium influx is not required for the establishment of this interaction.

**Figure 3:**
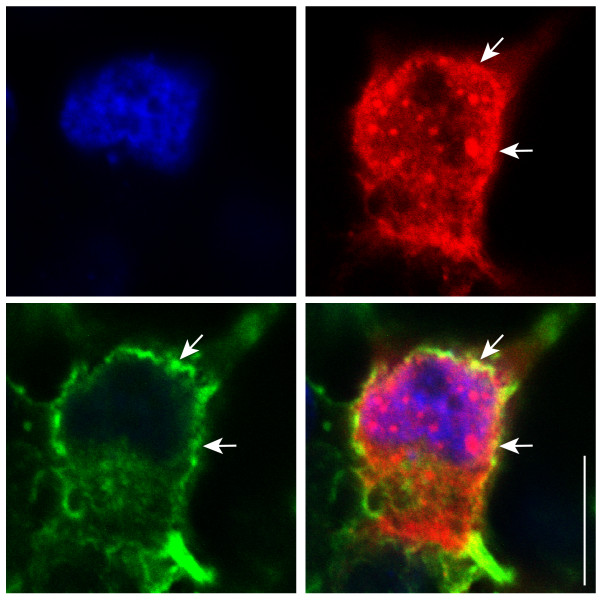
**Immunofluorescence localization of TRPC6 and SNF8.** M1R cells expressing FLAG-TRPC6 and HA-SNF8 were fixed and stained as follows: TRPC6 was detected with the anti-FLAG M2 mouse monoclonal antibody and an Alexa488 conjugated anti-mouse secondary (bottom left); SNF8 was detected with an anti-HA rabbit monoclonal antibody and a Cy3 conjugated anti-rabbit secondary (top right). Nuclei were counterstained with DAPI (top left). A merged image (bottom right) demonstrates partial localization of TRPC6 and SNF8 in punctuate structures (arrows). Bar = 10 μm.

To further confirm the potential interaction between SNF8 and TRPC6, the localization of the two overexpressed proteins was determined by immunofluorescence microscopy. Overall, there was little colocalization of the two signals, though small punctate regions suggesting areas of colocalization of the two proteins were seen in most cells examined (Figure 3).

### SNF8 enhances TRPC6-mediated current

**Figure 4:**
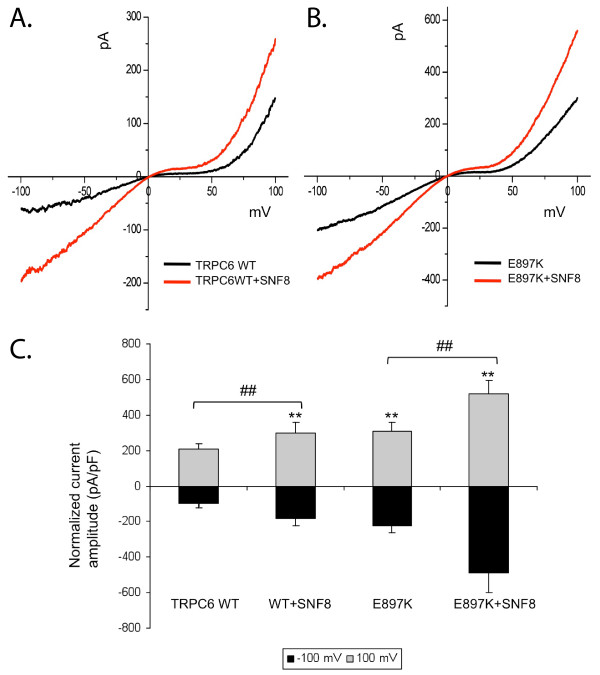
**SNF8 enhances TRPC6-mediated currents.** Whole-cell currents were measured in HEK cells expressing the M1 receptor and transiently transfected with either wild-type TRPC6 (**A**) or E897K mutant TRPC6 (**B**), alone (black line) or with SNF8 (red). Cells were stimulated with 100 μM carbachol to activate TRPC6-mediated current. **C**. Average normalized current density in response to carbachol. One way ANOVA Fisher LSD; ** p value <0.01 vs. TRPC6 WT, ## p value <0.01 between SNF8 and non-SNF8 expressing cells; n=6.

To address whether SNF8 may play a role in the regulation of TRPC6 activity, we performed electrophysiology experiments in the whole cell configuration in HEK293T cells expressing the muscarinic M1 acetylcholine receptor and either TRPC6 and SNF8, or TRPC6 alone. Activation of the M1 receptor with carbachol resulted in a whole cell current with a characteristic current-voltage relationship for TRPC6: a small inward and a steeply outwardly rectifying component. Co-expression of SNF8 led to increased whole cell current amplitude in both the inward and outward directions (Figure 4A and C). The ability of SNF8 to enhance TRPC6-mediated currents extended to channels containing an FSGS-associated mutation, E897K (Figure 4B and C). This mutation was previously characterized as a gain-of-function mutation leading to increased whole cell current amplitude compared to wild-type controls [15].

**Figure 5:**
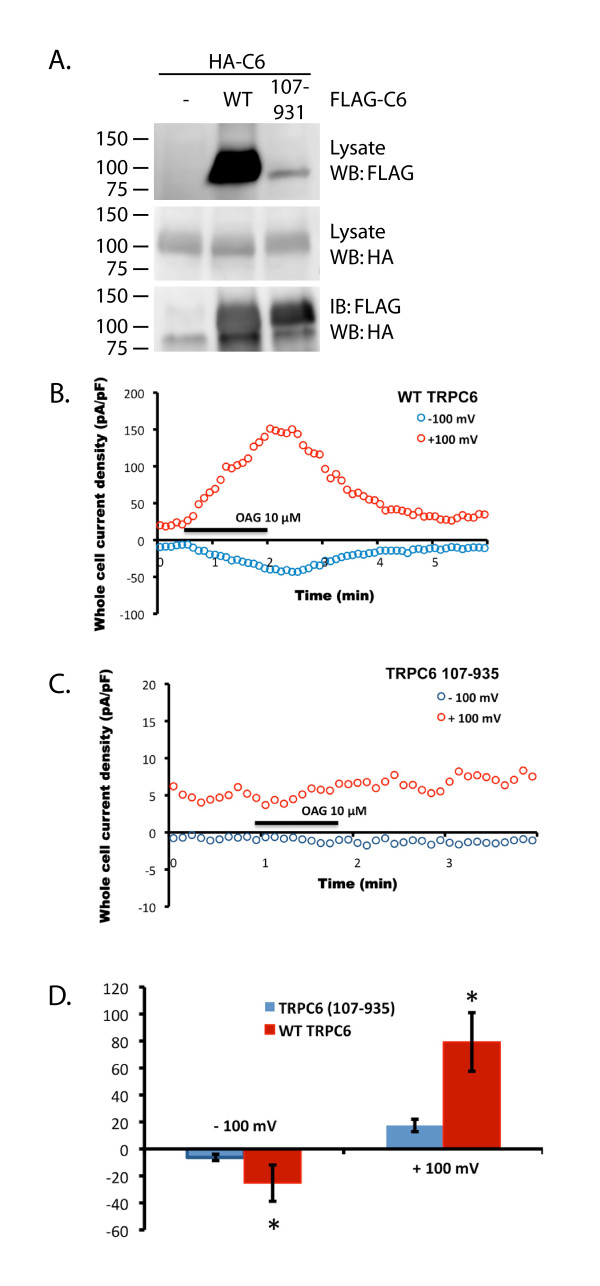
**Amino acids 1-106 are necessary for TRPC6 channel activity. A**. Full-length HA-tagged TRPC6 was expressed together with the indicated FLAG-tagged TRPC6 constructs in HEK 293T cells. Expression of FLAG- and HA-tagged protein was confirmed by Western blot of whole cell lysates (top and middle panels). The ability of the amino-terminal truncated TRPC6 protein to multimerize with full-length TRPC6 was confirmed by FLAG immunoprecipitation followed by western-blotting for HA-TRPC6 (bottom panel). Whole cell currents in HEK cells transiently transfected with wild-type TRPC6 (**B**) or TRPC6 107-931 (**C**) under basal conditions and upon stimulation with 10 μM OAG. **D**. Average normalized current density in response to OAG. One way ANOVA ; * p value < 0.05; n=8 cells.

We attempted to ascertain whether the effect of SNF8 on TRPC6 was dependent on direct binding of the two proteins. A TRPC6 construct lacking the amino terminal 106 amino acids (FLAG-TRPC6 107-931), required for SNF8 binding in the two hybrid assay, expressed at significantly lower levels than full-length TRPC6, though it was capable of forming multimers, based on its ability to co-immunoprecipitate full-length HA-tagged TRPC6 (Figure 5A). However, in contrast to full length TRPC6, the truncated protein failed to generate current in response to OAG stimulation (Figure 5B-D), preventing us from addressing whether the effect of SNF8 on TRPC6 currents was dependent on binding at this site. Similarly, TRPC6 107-931 failed to generate significant current after stimulation with carbachol (data not shown).

### Modulating SNF8 levels alters NFAT-mediated transcription downstream of mutant TRPC6

**Figure 6:**
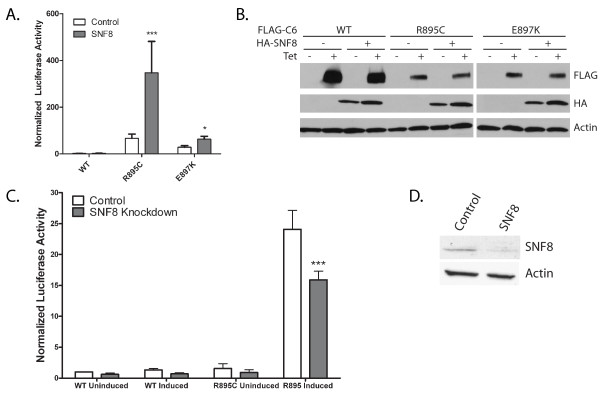
**SNF8 effect on NFAT-mediated transcription. A**. Cells stably expressing tetracycline-inducible wild-type, R895C or E897K TRPC6 were transfected with an NFAT-responsive luciferase reporter plasmid and either control plasmid or HA-SNF8 expression plasmid. Normalized luciferase activity was determined after TRPC6 expression was induced for 24 hours. Results are means ± SEM; one way ANOVA; * p<0.05 vs. control; ***p<0.001 vs. control. **B**. Western blots to confirm the expression of FLAG-TRPC6 and HA-SNF8 in lysates from cells used in the luciferase assays in (**A**). Actin was utilized as a loading control. **C**. Cells expressing wild-type or R895C TRPC6 under a tetracycline inducible promoter were transfected with plasmid encoding for either SNF8 shRNA (SNF8 Knockdown) or a scrambled shRNA (control), and luciferase reporter plasmids. 24 hours after transfection, TRPC6 expression was induced by the addition of tetracycline as indicated (induced). Cells cultured in the absence of tetracycline (uninduced) did not express detectable amounts of TRPC6, and were used as controls. 24 hours post-induction, luciferase reporter assays were performed. Results are means ± SEM; one way ANOVA, ***p<0.001 vs control shRNA. **D**. M1R cells were transfected with plasmids encoding for either an shRNA targeting SNF8, or a scrambled shRNA (control), as well as GFP. 48 hours post-transfection, cells were trypsinized and GFP expressing cells isolated by FACS cell sorting. Lysates from GFP positive cells were analyzed by Western blot for expression of SNF8. Actin expression was used as a loading control.

We have previously demonstrated that several FSGS-associated mutations in TRPC6 lead to activation of calcineurin-NFAT signaling and NFAT-mediated transcription in the absence of exogenous stimuli [38]. In light of the ability of SNF8 to enhance TRPC6-mediated whole cell currents, we examined the ability of SNF8 overexpression to affect TRPC6 mediated activation of NFAT-mediated transcription (Figure 6A). Overexpression of SNF8 in cells expressing either the R895C or E897K mutant TRPC6 channels led to an increase in NFAT-mediated transcription under unstimulated conditions. In contrast, in cells expressing wild-type TRPC6, which does not activate NFAT-mediated transcription under unstimulated conditions, SNF8 overexpression did not lead to activation of NFAT-driven transcription. Unfortunately, known activators of TRPC6, including carbachol and OAG, are able to substantially increase NFAT-mediated transcription in 293T cells even in the absence of exogenously expressed TRPC6 [38]. Therefore, we have not been able to ascertain whether overexpression of SNF8 is able to enhance NFAT activation by wild-type TRPC6 after the channel has been activated. Taken together with the electrophysiologic data, these results suggest that SNF8 can enhance TRPC6-mediated currents and downstream signaling pathways, but is not sufficient to activate the channel.

Most of our current knowledge regarding SNF8 relates to its function as part of the ESCRT-II complex involved in transmembrane protein trafficking into multivesiculated bodies [37,39,40]. We therefore hypothesized that overexpression of SNF8, rather than increasing ESCRT-II activity, might act in a dominant negative fashion by providing monomeric SNF8 to compete with intact ESCRT-II complex for TRPC6 binding. To address this possibility, we made use of a short hairpin RNA expressing plasmid to knock down SNF8 protein levels in M1R cells. Introducing the SNF8 shRNA expressing plasmid into cells expressing R895C mutant TRPC6 led to a modest decrease in activity from an NFAT-responsive luciferase reporter construct, compared to cells transfected with a control shRNA plasmid (Figure 6C). Expression of the SNF8 shRNA did not significantly alter basal NFAT-mediated transcription in control cells (WT uninduced and R895C uninduced) or expressing wild-type TRPC6 (WT induced). Western blot analysis confirmed a decrease in endogenous SNF8 levels with the SNF8 shRNA expression plasmid compared to control (Figure 6). These results suggest that: 1. the effects of overexpressed SNF8 on TRPC6 channel activity and signaling are not mediated by a dominant negative effect, and 2. while SNF8 may enhance TRPC6 activity, it is likely not required for channel activity given the only modest decrease in NFAT-reporter activity in response to SNF8 knockdown in R895C expressing cells. Confirmation of this later point will require the development of cells completely devoid of SNF8.

### SNF8 overexpression does not alter TRPC6 content on the cell surface or in detergent resistant membranes

**Figure 7:**
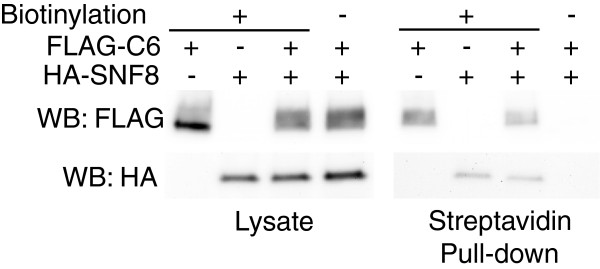
**Surface expression of TRPC6.** HEK293T cells were transiently transfected with the indicated combinations of FLAG-TRPC6 (FLAG-C6) and HA-SNF8 expression plasmids. 48 hours after transfection, cells were surface biotinylated or mock biotinylated (-) as indicated and lysed. Biotinylated protein and associated proteins were isolated by binding to streptavidin agarose beads. The amounts of TRPC6 and SNF8 in whole lysate (left panel) and bound to streptavidin beads (right panel) were assessed by Western blotting.

Several TRP channels, including TRPC6, have been reported to be regulated by altering their cell surface expression through regulated endo- and exocytosis [13,41,42]. Mutations in snf8 have been reported to affect the transport kinetics of Ena1p, a sodium pump in yeast [43]. We therefore wished to examine whether SNF8 overexpression might alter TRPC6 function by modulating the amount of channel expressed on the plasma membrane. M1R cells were transiently transfected with expression plasmids for FLAG-TRPC6 and HA-SNF8, either in combination, or alone with control plasmid. After surface biotinylation of the cells, the amount of TRPC6 bound to streptavidin beads (representing cell surface associated protein) was compared to total TRPC6 in whole cell lysates (Figure 7). Co-expression of SNF8 did not appreciably alter the amount of TRPC6 bound to the streptavidin beads compared to total TRPC6 levels. Interestingly, small amounts of SNF8 could also be detected in material bound to streptavidin beads (lower panels), consistent with its recruitment to transmembrane proteins, including plasma membrane proteins. However, the amount of SNF8 pulled down by streptavidin beads was not appreciably altered by the presence or absence of TRPC6, suggesting that cell surface proteins other than TRPC6 were largely responsible for this effect.

**Figure 8:**
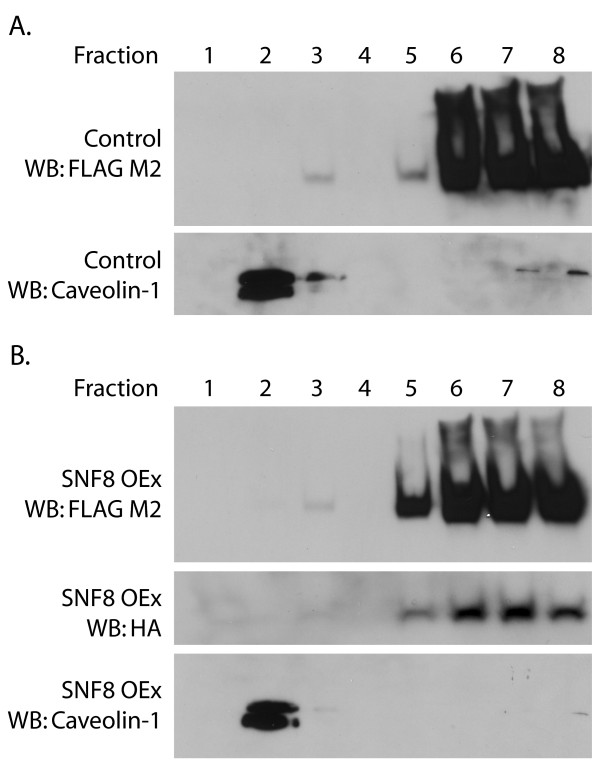
**Detergent-resistant membrane preparation.** M1R cells stably expressing TRPC6 were transiently transfected with control vector (**A**) or HA-SNF8 expression construct (**B**). Triton X-100 detergent resistant membranes were separated from other proteins by sucrose gradient centrifugation, and the abundance of FLAG-TRPC6 and HA-SNF8 in each fraction analyzed by Western blot. Caveolin-1 represents a marker of lipid rafts. Fraction 1 represents the top of the gradient.

Several TRPC proteins have been reported to partition into detergent-resistant membranes [44-50], and TPRC1 binds to caveolin-1, a lipid raft-associated protein [45,47-49,51-53]. Furthermore, STIM1 overexpression was reported to shift TRPC1 into lipid rafts, thereby altering its channel properties [52,54,55]. We therefore examined whether SNF8 may alter the amount of TRPC6 present in detergent-resistant membranes. In control cells, only a very small fraction of TRPC6 (Figure 8, top panel) was detected in the fractions containing detergent-resistant membranes, as assayed by the presence of the lipid raft marker caveolin-1 (Figure 8A, bottom panel), similar to previously reported results [46,50]. The amount of total TRPC6 found in the low density fractions did not change appreciably when HA-SNF8 was overexpressed (Figure 8B). A small amount of SNF8 was also detected in the detergent resistant membranes (Figure 8B, middle panel). It remains unclear why caveolin-1 localization peaked in fraction 2, while TRPC6 and SNF8 in the low density fractions were mainly in fraction 3, but the generation of distinct lipid raft subfractions has been reported using a protocol similar to the one used here [56].

## Discussion

Here we identify SNF8 as a novel binding partner of TRPC6. The interaction was first established in a yeast two-hybrid screen, where it was found to be mediated by the amino terminal 107 amino acids of TRPC6. The TRPC6-SNF8 interaction has been confirmed by co-immunoprecipitation, and the proteins partially colocalize in cells. Importantly, SNF8 overexpression enhances whole cell current amplitudes mediated by both wild-type and FSGS-associated mutant TRPC6, as well as mutant TRPC6-mediated NFAT activation, while RNAi mediated knock-down of endogenous SNF8 diminishes NFAT activation by mutant TRPC6. The effect of SNF8 on TRPC6 channel activity and downstream activation of NFAT-mediated transcription does not appear to be mediated by alterations in the global surface expression levels of TRPC6 or a shift of channel into lipid rafts. Taken together, these results suggest a novel role for SNF8 in the modulation of TRPC6 channel activity.

It is difficult to reconcile the ability of SNF8 to enhance TRPC6-mediated current with its major known function, sorting transmembrane proteins into multivesiculated bodies for degradation. SNF8, together with VPS25 and VPS36, forms the endosomal sorting complex for transport (ESCRT) -II complex [35]. ESCRT-II, in conjunction with the ESCRT-I and –III complexes, is involved in the budding of intraluminal vesicles from endosomes to form multivesiculated bodies (MVB) (reviewed in [37,39,40]). This process is critical for the downregulation of multiple signaling receptors, including the yeast mating receptor Ste2 [35], EGFR[36,57] and Notch [58,59]. The ESCRT complexes not only target ubiquitinated transmembrane proteins to the vesicles of the MVB, but induce the necessary membrane curvature and ultimate scission of the vesicles from their parent membrane [60-62]. In addition to their role in MVB formation, ESCRTs are involved in the membrane scission required for cytokinesis and the budding of enveloped viruses [63,64].

Whether the endosomal-lysosomal functions of SNF8 might relate to TRPC6 activity is unclear. It is worth noting that SNF8 has been implicated to have several non-endosomal functions. These include the establishment of the bicoid mRNA gradient in the *Drosophila* oocyte [65] and modulation of the ELL transcription elongation complex [66]. Perhaps most relevant to its role in enhancing TRPC6-mediated currents, the yeast homologue of SNF8, VPS22, as well as other members of the ESCRT complexes, have been shown to be involved in the trafficking and surface expression of the sodium pump Ena1 [43]. Although we have not been able to detect a change in the amount of total TRPC6 expressed on the cell surface or in lipid rafts in response to co-expression of SNF8, it is possible that SNF8 traffics the channel to a subdomain of the plasma membrane where TRPC6 activity is enhanced. Along these lines, it is noteworthy that podocin enhances TRPC6 activity in a cholesterol dependent manner without altering plasma membrane expression [14], while the differential requirement of ESCRT-II for the budding of avian sarcoma and leukosis virus (ASLV) and human immunodeficiency virus, type-1 (HIV-1), correlates with their assembly on phosphatidylethanolamine (PE) containing or PE-negative membranes, respectively [63,67]. Alternatively, it is possible that SNF8 regulates TRPC6 indirectly by competing with a negative regulator or by altering the activity of another membrane protein in the vicinity of TRPC6, such as an enzyme that affects phosphoinositide levels. Finally, TRPC6 has been reported to be activated by membrane deformation [68], though this has been disputed [69]. One could hypothesize that SNF8 may act to recruit ESCRT-II to TRPC6, and alter TRPC6 function through local convex deformation of the membrane [70]. Further understanding the mechanism whereby SNF8 enhances TRPC6 currents will be the goal of future investigations.

## Conclusion

This work shows that: 1. SNF8 is a potential binding partner of TRPC6, 2. overexpression of SNF8 enhances both wild-type and mutant TRPC6 current densities, and 3. modulating SNF8 expression levels affects NFAT activation downstream of gain-of-function, FSGS-associated TRPC6 mutations. The mechanism for regulating channel activity is not mediated by changes in global cell surface expression or recruitment into lipid rafts. Taken together, these results identify SNF8 as a potent modulator of the TRPC6 channel.

## Methods

### Plasmids and reagents

The human TRPC6 coding sequence, with or without mutations as outlined in the text, and containing an amino-terminal FLAG tag sequence, was cloned into pcDNA4/TO/myc-HIS B (Clontech) using standard PCR-based techniques. Similarly, full-length human TRPC6 carrying an amino-terminal HA tag was amplified by PCR and subcloned into pcDNA3.1. The HA-SNF8 expression plasmid was a gift from C. Bucci [71]. The Matchmaker Two-Hybrid System and *S. cerevisiae* Y187 pre-transformed with a human kidney cDNA library were purchased from Clontech (Palo Alto, CA). The dual luciferase assay kit and reporter vectors pGL4.30 and pGL4.74 were obtained from Promega. Affinity purified rabbit anti-TRPC6 polyclonal antibody was purchased from Chemicon, anti-FLAG M2 monoclonal antibody and anti-FLAG rabbit polyclonal antibodies were purchased from Sigma, rabbit anti-GFP polyclonal antibody and mouse anti-HA monoclonal antibody were purchased from Abcam Inc, and rabbit anti-HA monoclonal antibody (C29F4) was purchased from Cell Signaling Technologies. Anti-SNF8 rabbit polyclonal antibody was the kind gift of Dr. H. Stenmark [36]. Anti-caveolin-1 mouse monoclonal antibody (clone 2297) was obtained from BD Biosciences.

### Yeast two-hybrid screen

cDNA encoding residues 1 through 406 of TRPC6 (wild-type N-terminal domain) was used as bait and cloned in-frame with GAL4 DNA-binding domain in the vector pGBKT7-BD and transformed into yeast strain AH109. The bait strain was mated to Y187 yeast strain pre-transformed with a commercially available human kidney cDNA library cloned into pACT2-AD vector according to the manufacturer’s protocol (Clontech). Based on mating efficiency, 1 × 10^6^ clones were screened. Mated yeast cells were grown on high-stringency selection plates (SD-Leu, Trp, Ade, His) and positive colonies were further verified by growth on high-stringency plates supplemented with X-α-gal as a test for β-galactosidase activity. pACT2-AD plasmids containing library inserts from positive colonies were isolated and transformed into DH5α derived *E. coli* (New England Biolabs). Plasmids were then isolated from bacteria, sequenced, and analyzed using the Blast-NT alignment algorithm from NCBI.

To confirm the interaction, the full-length SNF8 coding sequence was cloned inframe into the pGAD-T7 plasmid and the resulting plasmid transformed into Y187 strain yeast. The resulting strain was mated with AH109 bait strain harboring pGBKT7 with wild-type TRPC6 N-terminal sequences or the TRPC6 C-terminal domain (corresponding to amino acids 726-931). The resulting diploids were tested for positive interaction by growth on high-stringency plates.

### Cell culture and luciferase assays

Cells stably expressing the M1 muscarinic acetylcholine receptor and either wild-type, R895C or E897K TRPC6 under a tetracycline-inducible promoter were generated from T-Rex-293 cells (Invitrogen), as previously described [38]. Stable cell lines were induced to express TRPC6 for 24 hours prior to lysis by adding tetracycline to a 1 μg/ml final concentration in culture medium. Luciferase assays were carried out essentially as previously described [38] using the dual luciferase reporter assay (DLR; Roche) using a Veritas microplate luminometer (Turner Biosystems). All conditions were tested in triplicate and results normalized to the Renilla luciferase internal control.

### RNA interference

SNF8 was knocked down using short hairpin-expressing double-stranded oligonucleotides cloned into pcDNA 6.2-GW/EmGFP-miR plasmids (BLOCK-iT™ Pol II miR RNAi Expression Vector Kit, Invitrogen). A specific shRNA sequence against SNF8 (sense 5′-TGACTTCG[CC]CAATGTCAGTCAA-3′ and antisense 5′-TTGACTGACATCCTGGGCGAA-3′), and a scrabbled control sequence, were obtained from Invitrogen and cloned into the plasmid. Knock-down efficiency was determined by transient transfection of M1R cells with either SNF8 or control plasmid, followed by FACS sorting for GFP expression to isolate the transfected cells after 48 hrs. SNF8 expression in sorted cells was assessed by immunbloting for SNF8.

To assess the effect of SNF8 knock-down on NFAT-mediated transcription, cells were transfected with the indicated shRNA expression vector and luciferase reporter plasmids using Fugene 6. 24 hours after transfection, TRPC6 expression was induced where indicated by the addition of tetracycline (1μg/ml final concentration) to the media. After an additional 24 hours, cells were lysed and processed for dual luciferase reporter assay.

### Immunoprecipitation and Immunoblotting

Transfected cells were rinsed once in phosphate buffered saline and lysed in modified RIPA lysis buffer (50 mM Tris, pH 8.0, 150 mM NaCl, 1% NP-40, 0.5% sodium deoxycholate supplemented with Complete protease inhibitor cocktail (Roche)). After clearing lysates by centrifugation at 14,000 RPM for 15 minutes at 4ºC, supernatants were incubated with 30 μl of FLAG M2 agarose slurry (Sigma-Aldrich), and incubated with constant agitation at 4ºC for 2-3 hours. Immunoprecipitated complexes were washed three times with lysis buffer and eluted off of the beads by boiling in SDS-sample loading buffer.

Cell lysates and immunoprecipitated materials were separated by SDS-PAGE and transferred to PVDF membrane (Bio-Rad). The membrane was blocked with 5% non-fat milk in PBST (PBS with 0.05% Tween-20) for 1 hour at room temperature, followed by overnight incubation in 1:1000 anti-TRPC6, 1:500 anti-HA, 1:500 FLAG M2, or 1:200 anti-SNF8 antibody in 5% non-fat milk PBST. After three washes in PBST, blots were incubated with the appropriate secondary antibody conjugated to HRP (Pierce) in PBST at room temperature, followed by detection with SuperSignal West Pico chemiluminescent substrate (Pierce).

### Fluorescence microscopy

Cells stably expressing wild-type TRPC6 under a tetracycline inducible promoter were grown on collagen I coated glass coverslips and transfected with plasmid encoding for HA-SNF8 using Fugene6 followed by treatment with tetracycline to induce FLAG-TRPC6. 24 hours after inducing TRPC6 expression, cells were washed with PBS, fixed in 2% paraformaldehyde, 4% sucrose in PBS, and permeabilized with 0.3% Triton X-100 in PBS. Nonspecific binding sites were blocked using blocking solution (2% bovine serum albumin, 2% fetal calf serum, 0.2% fish gelatin). Cells were incubated with 1:200 anti-FLAG M2 antibody and 1:1600 anti-HA rabbit monoclonal antibody, followed by Alexa488 conjugated goat anti-mouse and Cy3 conjugated goat anti-rabbit secondary antibody (Jackson ImmunoResearch), and mounted on slides with ProLong Gold antifade reagent (Invitrogen). Images were taken using a Zeiss LSM 510 confocal microscope.

### Electrophysiology

Patch-clamp electrophysiology (Axopatch 200B amplifier, Axon Instruments, CA) was performed in the whole-cell configuration. Briefly, HEK293T cells (American Type Culture Collection, VA) were plated on glass coverslips at low density and placed in the recording chamber. The patch pipettes with resistances of 3–4 MΩ were pulled from borosilicate glass with a P-97 puller (Sutter Instrument) and filled with a solution containing (in mM): 135 CH3SO3Cs, 10 CsCl, 3 MgATP, 0.2 NaGTP, 0.2 EGTA, 0.13 CaCl2, and 10 HEPES, pH 7.3 with CsOH. The bath solution contained (in mM): 135 CH3SO3Na, 5 CsCl, 2 CaCl2, 1 MgCl2, 10 HEPES, and 10 Glucose, pH 7.4 with NaOH. Carbachol (100 μM) or 1-oleoyl-2-acetyl-sn-glycerol (OAG, 10 μM) was applied to the bath solution. Whole-cell currents were recorded from –100 mV to 100 mV voltage ramps over 150 ms and a holding potential of 0 mV. All data were acquired at room temperature and analyzed using pClamp 10 (Axon Instruments, CA). Statistical analysis was done using one-way ANOVA Fisher’s LSD test, and p values <0.05 were considered significant.

### Surface biotinylation

Cells were surface biotinylated on ice using Sulfo-NHS-SS-biotin (Pierce) 48 hours after transfection, essentially as previously described [38], followed by lysis in modified RIPA buffer. Lysates were incubated with streptavidin beads (Pierce) at 4°C. The streptavidin beads were extensively washed and bound material was eluted and analyzed by SDS-PAGE electrophoresis followed by Western blot analysis.

### Lipid raft preparation

M1R cells stably expressing FLAG-tagged TRPC6 were transfected with control or HA-SNF8 expression constructs. 48 hours after transfection, cells were processed using a protocol modified from Alicia et al [54]. Briefly, cells were washed in ice cold PBS, scraped off of plates in PBS and lysed in 1% Triton X-100 in MES buffer (25mM MES, pH 6.5, 150mM NaCl, 2mM EDTA complemented with protease inhibitors) on ice for 20 minutes. Lysates were further homogenized in a glass homogenizer with the loose fitting piston, then mixed with an equal volume of 85% (w/v) sucrose in MES buffer. Lysates were overlayed with 4mls of 40% sucrose in MES buffer followed by 4mls of 5% sucrose in MES buffer. Lysates were spun at 175,000x g for 24 hours in an SW41 Ti rotor. Eight 1.5 ml fractions were obtained by aspiration starting at the top of the gradient. Aliqouts of each fraction were combined with 4x sample loading buffer with β-mercaptoethanol and analyzed by Western blot as above.

## Abbreviations

ASLV: Avian sarcoma and leukosis virus; ESCRT: Endosomal sorting complex for transport; FSGS: Focal segmental glomerulosclerosis; GFP: Green fluorescent protein; HA: Hemaglutinin; HIV: Human immunodeficiency virus; NFAT: Nuclear factor of activated T-cells; PAGE: Poly-accrylamide gel electrophoresis, PE, Phosphatidylethanolamine; shRNA: Short hairpin ribonucleic acid; TRPC: Canonical transient receptor potential.

## Competing interests

The authors declare that they have no competing interest.

## Authors’ contributions

RC performed the yeast-two hybrid screen and candidate identification, generated shRNA plasmids, performed SNF8 knockdown experiments and luciferase assays, and helped draft the manuscript. SK generated expression constructs, and performed cell transfections and immunoblotting. MRP participated in the design and coordination of the study. DT and AG performed all electrophysiology experiments and related statistical analysis, and helped draft the manuscript. JS conceived of the study, performed co-immunoprecipitation, immunofluorescence, surface biotinylation, membrane fractionation and luciferase assays, performed statistical analysis and drafted the manuscript. All authors read and approved the final manuscript.
